# Observation of Wavelength-Dependent Quantum Plasmon Tunneling with Varying the Thickness of Graphene Spacer

**DOI:** 10.1038/s41598-018-37882-z

**Published:** 2019-02-04

**Authors:** Khang June Lee, Shinho Kim, Woonggi Hong, Hamin Park, Min Seok Jang, Kyoungsik Yu, Sung-Yool Choi

**Affiliations:** School of Electrical Engineering, Center for Advanced Materials Discovery towards 3D Displays, Korea Advanced Institute of Science and Technology (KAIST), 291 Daehak-ro, Yuseong-gu, Daejeon 34141 Republic of Korea

## Abstract

Plasmonic coupling provides a highly localized electromagnetic field in the gap of noble metals when illuminated by a light. The plasmonic field enhancement is generally known to be inversely proportional to the gap distance. Given such a relation, reducing the gap distance appears to be necessary to achieve the highest possible field enhancement. At the sub-nanometer scale, however, quantum mechanical effects have to be considered in relation to plasmonic coupling. Here, we use graphene as a spacer to observe plasmonic field enhancement in sub-nanometer gap. The gap distance is precisely controlled by the number of stacked graphene layers. We propose that the sudden drop of field enhancement for the single layer spacer is originated from the plasmon tunneling through the thin spacer. Numerical simulation which incorporates quantum tunneling is also performed to support the experimental results. From the fact that field enhancement with respect to the number of graphene layers exhibits different behavior in two wavelengths corresponding to on- and off-resonance conditions, tunneling phenomenon is thought to destroy the resonance conditions of plasmonic coupling.

## Introduction

A surface plasmon is a quantum of the collective electron oscillation forming at the interface between dielectric and metallic materials or on noble metal nanoparticles (NPs). At a frequency of incident light that causes a resonance with the surface plasmon, light can be trapped on the surface of such NPs. Such a strong light-matter interaction has been employed to develop various applications, such as surface-enhanced Raman spectroscopy (SERS)^1,2^, high-harmonic generation^3^, and plasmonic photodetectors^4,5^ and photovoltaics^6^. One effective method for enhancing the localized field is to make neighboring NPs plasmonically coupled with each other. Each pair in this dimerized structure consists of two plasmonic NPs spatially separated by a dielectric, and thus strong near-fields are known to be formed around the NPs. Additionally, the field enhancement is also known to be strongly inversely proportional to the distance between NPs because of the presence of highly polarized electric field induced by the Coulomb interaction^7,8^. Hence, ways of having NPs packed closely together are generally considered to be important to achieve high field enhancement^9^.

If the gap distance becomes sub-nanometer scale, however, the field enhancement effect is rather substantially reduced because of the plasmon tunneling^10,11^. Such a tunneling phenomenon occurs through the potential energy barrier formed by the insulating material between two metals^12–14^. In this sense, understanding the characteristics of plasmonic coupling in sub-nanometer scale is important to pave the way for practical plasmonic applications. Thus far, various plasmonic structures with sub-nanometer gaps have been proposed to investigate the quantum tunneling effect^15–17^. In addition to the gap distance dependence, Hajisalem *et al*. showed that not only the geometrical dimensions of metallic structure itself but the refractive index of insulator between metallic structures also affects the onset of quantum tunneling effect^18^, which could be important for practical applications. In this way, although many researchers have devoted considerable efforts to studying the plasmon tunneling in the gap range of sub-nanometer scales, literatures that have considered its dependence relevant to plasmonic resonance characteristics are rarely seen. To properly analyze the quantum tunneling effect in plasmonic system, one important aspect that needs to be considered carefully is how the resonance condition changes with respect to changes in the gap distance between metallic structures.

In this paper, we study how the plasmon tunneling through sub-nanometer scale gaps affects the resonance condition associated with plasmonic coupling. A vertically stacked structure of gold-NPs/graphene/gold-film is used to investigate the gap distance-dependent plasmonic coupling, the magnitude of which can be determined by the SERS intensity. Graphene is considered to be appropriate to examine such dependence because its thickness can be easily controlled by simply changing the stacking number of single-layer graphene (SLG)^19^. Graphene is also considered to be an insulating spacer from the fact that not only its electrical resistance in the direction perpendicular to the layers is much higher than that of metallic structures^20–22^, but its vertical conductivity is also suppressed at optical frequencies^23^. The graphene layers in our structure have indeed turned out to play a key role as a spacer that can maintain the steady-state of plasmonic coupling between gold nanoparticles (Au-NPs) and their image charges formed in the gold film (Au-film)^20,24^.

In addition to the dependence on the graphene spacer thickness, we also investigate the wavelength-dependent plasmon tunneling. As the gap narrows, we have found that the resonance peak wavelength associated with the plasmon bonding mode steadily exhibits redshift and the wavelength-dependent field enhancement generally increases except for a single-layer thickness^13,15^. In the case of an SLG spacer, however, the resonance condition of plasmonic coupling is totally destroyed by the plasmon tunneling, and thus the field enhancement is considerably reduced in most spectral ranges. Such a tunneling phenomenon gradually disappears as the wavelength becomes large. This suggests that when designing plasmonic nanostructures a proper combination of spacer thickness and light wavelength needs to be carefully considered to achieve the highest possible plasmonic performance.

## Results

**Figure 1:**
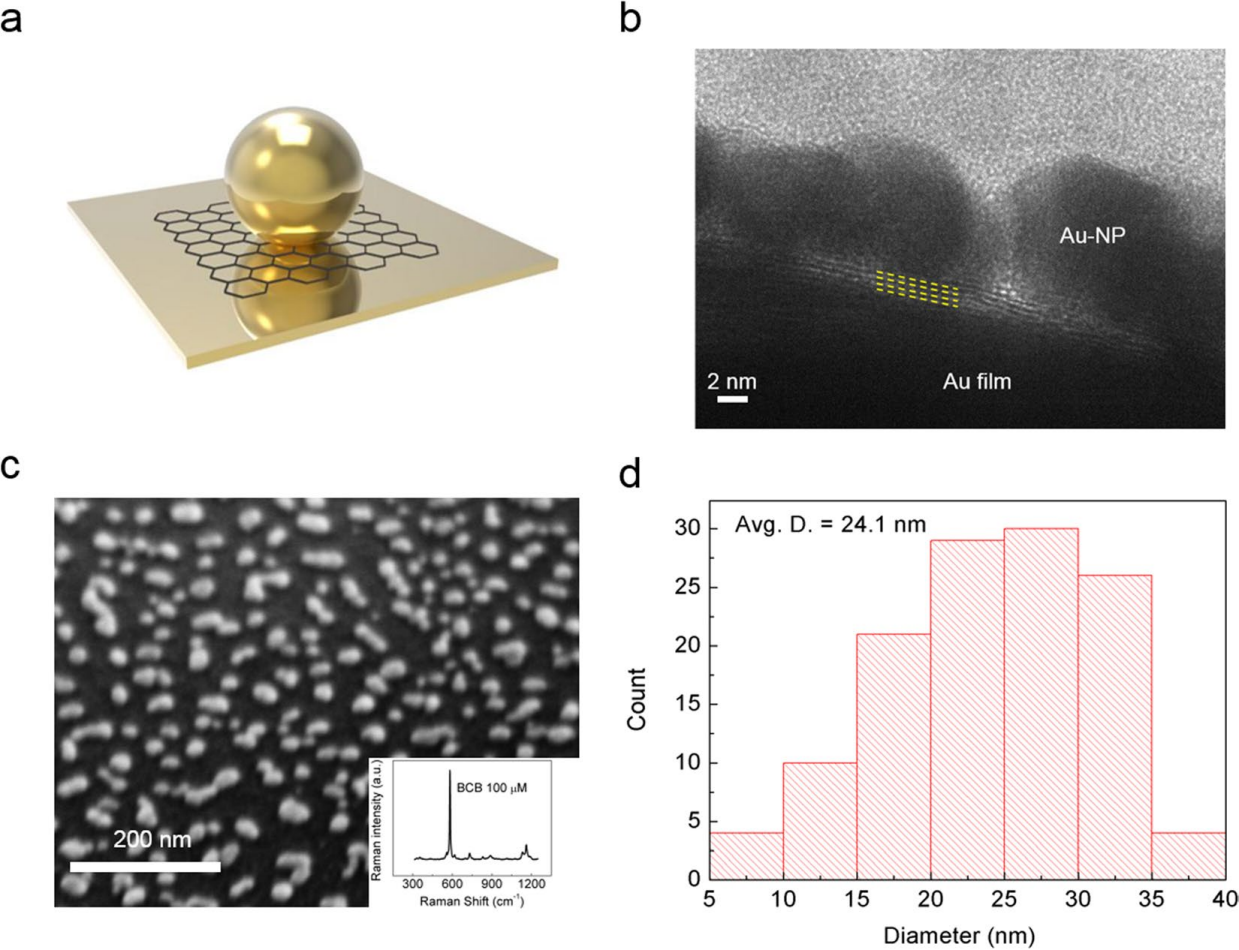
(**a**) Schematic illustration of the three-dimensional graphene spacer structure (NPs/graphene/film). The gap distance between the top NPs and a bottom metal film can be precisely and deterministically controlled by the number of graphene layers. (**b**) TEM image of the structure. Four layers of graphene (denoted with yellow dashed lines) are clearly seen without showing any inter-layer residues. (**c**) SEM image of randomly distributed Au-NPs on top of the graphene/Au film stack. Inset shows the Raman spectrum of BCB which was used as a test molecule of SERS. (**d**) Histogram showing the distribution of Au-NPs diameters based on several SEM images. The average diameter is about 24.1 nm.

Figure 1a shows a schematic of the graphene spacer structure fabricated as a vertical stack of Au-NPs/graphene/Au-film. Taking advantages of the discrete nature of the SLG transfer process, we are able to vary the thickness of the spacer from one to four layers of graphene, by repeating the SLG transfer for each of the desired number of graphene layers. The thickness of the spacer is determined by the number of stacked layers multiplied by the thickness of an SLG, 0.34 nm^25^. The average diameter of Au-NPs was found to be approximately 24.1 nm (Fig. 1d). To produce SERS test molecules, a solution of brilliant cresyl blue (BCB) molecules with a concentration of 100 μM was drop-casted and dried at room condition. A typical Raman spectrum from BCB molecules is shown in the inset of Fig. 1c. The details of the experimental set-up can be found in Experimental Section.

A cross-sectional transmission electron microscopy (TEM) image of the graphene spacer layer is shown in Fig. 1b. A four-layer graphene film without any inter-layer residue can be clearly seen in the layer-by-layer multi-stacked structure. The gap distance between the Au-NPs and the Au film is approximately 1.36 nm, which corresponds to the thickness of an SLG multiplied by the number of graphene layers (four). A scanning electron microscope (SEM) image of the Au-NPs on a graphene spacer is shown in Fig. 1c. The observation angle was tilted by 54° to highlight both the approximately spherical shape of the NPs and the smoothness of the bottom metal film. The Au-NPs are distributed in the lateral dimension with an average separation distance of approximately 40 nm. This lateral separation is significantly large compared to the vertical spacer thicknesses (up to four layers of graphene). This implies that the lateral plasmonic coupling between adjacent Au-NPs can be ignored (see Supplementary Fig. 2 for details). In other words, most near-field enhancement in the structure studied here originates from the vertical plasmonic coupling between the top Au-NPs and their image charges mirrored in the bottom Au film.

**Figure 2:**
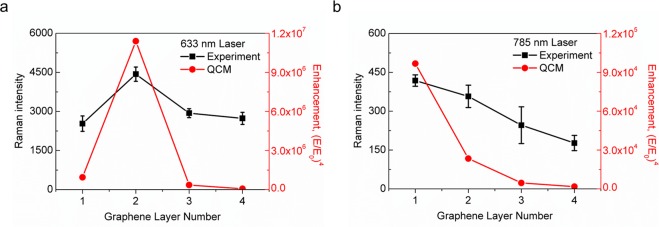
Comparison between the experimental (black) and simulated (red) optical responses for samples excited by (**a**) a 633 nm laser and (**b**) a 785 nm laser. The left vertical axis represents the peak Raman intensity observed from the SERS spectra by using BCB molecules. The right vertical axis indicates the estimated signal enhancement (E/E_0_)^4^ obtained from the finite element method (FEM)-based electromagnetic simulations with consideration of the QCM effects. For 633 nm case, both the Raman intensity and the enhancement generally increase as the number of graphene layers decreases, except the SLG spacer in which both values drop suddenly. In contrast, for samples illuminated by a 785 nm laser, both values keep increasing even down to a single-layer graphene case.

Figure 2 compares the experimental results and the quantum-corrected model (QCM)-based numerical simulations^26^. The QCM incorporates quantum tunneling within a classical electrodynamics framework, and closely agrees with other quantum mechanics-based simulations. To consider the plasmon tunneling, we introduce a QCM channel in a numerical simulation to represent the corresponding tunneling resistances in the gap region (see Supplementary Fig. 1). The field enhancements calculated in the simulation are obtained in the region near the junction between Au-NPs and the surface of graphene. The experimental results are based on the peak intensity observed in the SERS spectrum measurement which is proportional to the fourth order of the relative enhanced field (E/E_0_). The left vertical axes in Fig. 2 represent the Raman intensity observed by the SERS signals, while the right vertical axes indicate the enhancement of (E/E_0_)^4^ obtained from the QCM simulation. The original Raman spectra of BCB molecules for each layer are shown in Supplementary Fig. 3.

For samples excited by a 633 nm laser, both the Raman intensities and the calculated enhancement show very similar trends: increasing as the number of graphene layers decreases to two, with a sudden drop at the single layer. The exponentially increasing trend with the decreasing thickness can be explained by classical electromagnetic theory. In contrast, the sudden drop of the near-field enhancement for the SLG case implies that the classical approach is no longer valid in this thin barrier. Considering that the QCM simulation is consistent with the experimental result even for the SLG case, the sudden drop is considered to be evidence of plasmon tunneling. The finite probability of electron tunneling through such a thin spacer is expected to reduce the field intensities across the spacer gap, resulting in a significantly decreased near-field enhancement. The thickness of an SLG spacer (0.34 nm) is sufficiently thin to initiate plasmon tunneling, as reported in previous works^12^. We also note that the work function of graphene (approximately 4.6 eV) is comparable to that of most noble metals. Therefore, tunneling through a graphene spacer may be much more probable than tunneling through a vacuum spacer with the same thickness because of its smaller barrier height^18,27^. We have found that the relative permittivity of the graphene spacer undergoes a dramatic change in its real part as its thickness decreases. Supplementary Fig. 4 shows such a thickness-dependent permittivity employed in the QCM simulation. As the thickness decreases to approximately 4 Å, the real part of the relative permittivity begins to decrease, and then finally reaches a negative value. Therefore, the channel that is initially insulating in the large thickness changes to exhibit metallic behavior at smaller thicknesses because of the active plasmon tunneling.

**Figure 3:**
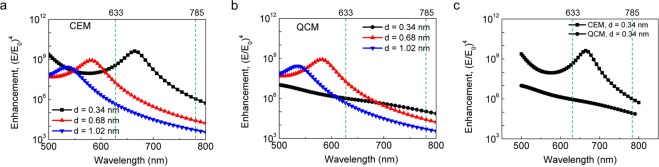
Simulated field enhancement with respect to the input wavelength. (**a**) CEM results, and (**b**) QCM results. The vertical dashed lines indicate the two wavelengths used for the simulation. For a double- or a triple-layer graphene, the curves obtained from both methods show the plasmon resonance peaks and they are essentially the same. For a single-layer graphene, however, the plasmon resonance peak is still present when the CEM method is used while its corresponding peak does not exist in the whole range of 500–800 nm when the QCM method is used. (**c**) Comparison between CEM and QCM simulations for the single-layer case. Compared with the enhancement obtained from the CEM, the relatively small enhancement from the QCM can be attributed to the quantum tunneling through the thin spacer.

An interesting feature can be found by comparing Fig. 2a,b. For samples excited by a 785 nm laser, both Raman intensities and enhancement obtained by the QCM simulation (Fig. 2b) show similar trends as in the 633 nm case. However, the 785 nm case does not show a sudden drop at an SLG spacer. The discrepancy between the 633 nm and the 785 nm cases can be explained by the resonance breaking through the plasmon tunneling in the 633 nm excitation. Figure 3a shows the enhancement obtained from the classical electromagnetic model (CEM) simulation under various incident wavelengths, reflecting resonance behavior between light and the graphene spacer structure. As the gap narrows, the resonance peak undergoes a redshift. For the QCM simulation shown in Fig. 3b, however, the resonance peak for an SLG spacer does not exist, whereas the peaks for a double- and a triple-layer spacer remain unchanged. This absence of the resonance, hereinafter called resonance breaking, is due to the plasmon tunneling through the SLG spacer layer. The electromagnetic field is tightly confined between Au-NP and bottom Au substrate due to the antisymmetric charge distribution for y-axis as shown in Supplementary Fig. 5(a). Once the tunneling phenomenon occurs, the gap region of graphene spacer appears to have metallic properties and Au-NPs and their image charges are electrically connected by forming a vertical dumbbell-like structure. Thus the field enhancement near the gap is severely reduced. Moreover, the plasmon bonding mode is known to change from a mirror-induced bonding dipolar plasmon (MBDP) mode to charge transfer plasmon (CTP) mode^28^. Because of this mode change from MBDP to CTP, the resonance peak at approximately 680 nm shown in the CEM simulation disappears in the QCM simulation (see Fig. 3b, black line). Therefore, the resonance breaking results in different field enhancement behavior for 633 nm and 785 nm excitations. By comparing a single- and a double-layer spacer in Fig. 3b, the magnitude of their enhancements is observed to be reversed at approximately 700 nm. As a result, the enhancement associated with the 633 nm illumination exhibits a sudden drop with an SLG spacer, whereas that with the 785 nm illumination reaches its highest value in the SLG case.

Another explanation can be considered from the perspective of the wavelength dependency of plasmon tunneling. Figure 3c shows the CEM and QCM simulations for an SLG spacer. The enhancement difference (CEM – QCM), namely the enhancement obtained from the QCM subtracted from that from the CEM, indicates the magnitude of the tunneling current because the QCM enhancement is reduced from the CEM enhancement by the presence of the tunneling current. The plasmon tunneling is indeed strongly affected by the wavelength of incident light. For example, as shown in Fig. 3c, the enhancement difference is much larger with 633 nm excitation than with 785 nm excitation, meaning that the tunneling is much more active at a resonance wavelength in the vicinity of 633 nm than at an off-resonance wavelength of 785 nm. This significant tunneling in the 633 nm case is considered to result in the relatively small enhancement observed in experiments with an SLG spacer. If the tunneling is independent of the wavelength (i.e. direct quantum tunneling in direct current bias), the sudden drop in the enhancement will also be observed in the 785 nm case. As the wavelength becomes longer, the CEM curve gradually approaches the QCM curve, which means that the tunneling becomes weaker with the longer wavelength. With sufficiently long wavelengths, the plasmon tunneling is expected to no longer occur even with an SLG spacer and the whole system can be considered to be outside the quantum regime. To observe the plasmon tunneling with the long wavelength, a much smaller thickness of the spacer is needed to induce the large redshift of the resonance peak. If the redshifted resonance peak matches the wavelength of incident light, plasmon tunneling will occur through the very small gap. In a sub-nanometer spacer, the magnitude of redshift is known to be accelerated with narrower spacer^29^ and the resonance peak finally disappears because of plasmon tunneling^13^. Therefore, studies on plasmon tunneling and on achieving the highest possible enhancement in quantum plasmonics indicate that not only is careful attention needed when designing a nanostructure, but a favorable wavelength must also be selected.

Although the experimental results for samples illuminated by a 633 nm or a 785 nm laser are generally in good agreement with the QCM simulation as shown in Fig. 2a,b, both results are not perfectly matched with each other. These small deviations are attributed first to the fact that the Raman intensity can be affected by the size of the Au-NPs used in the experiment. As shown in Supplementary Fig. 6, different particles sizes lead to differences both in the field enhancement and in the location of the resonance peaks. As the diameters of the NPs increase, not only does the resonance peak undergo red-shift, but the field enhancement also becomes higher. Raman intensity is obtained from a statistical characterization of the BCB molecules that are situated in the area illuminated by a laser. Within a laser spot size of approximately 1 μm, a large number of NPs with various diameters respond to the plasmonic coupling. The deviations between the experimental and numerical results can also be attributed to our assumption that each NP has a perfectly spherical shape in the simulation. Many NPs that are evaporated on a spacer indeed have non-spherical irregular shapes because of wetting on the graphene surface as shown in Fig. 1c. Other possible reasons such as laser light scattering, defects in graphene, and Stokes shift^30^ can also be considered in a real experimental situation, although these play minor roles.

**Figure 4:**
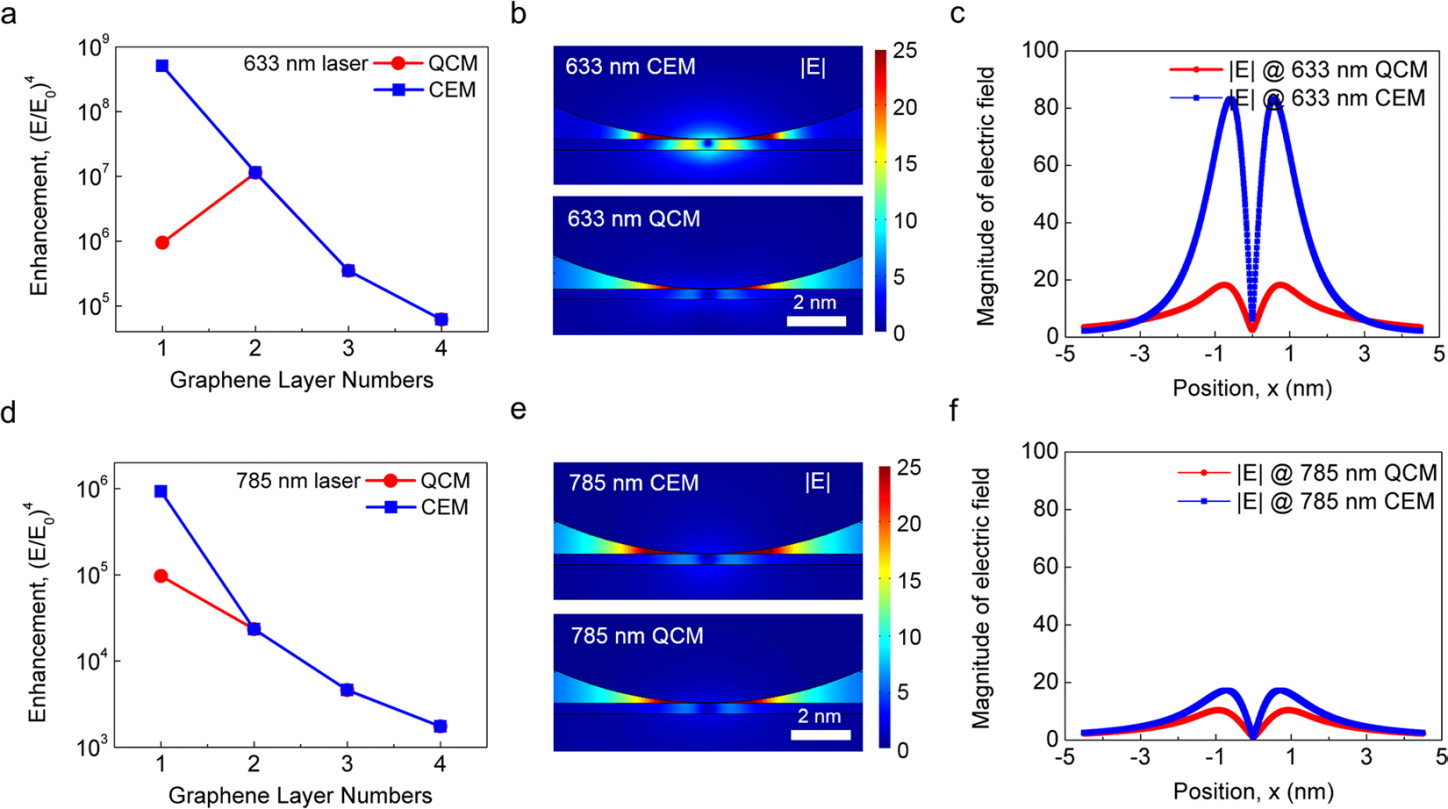
Comparison between two simulations: QCM and CEM. Shown in the upper and lower rows of the panels are the results for 633 nm and 785 nm excitation, respectively. (**a**,**d**) The samples with a multi-stacked graphene spacer (2–4 layers) show almost the same enhancement for both QCM and CEM. For a sample with an SLG spacer, however, the enhancement simulated by QCM is quite smaller than that obtained by CEM. (**b**,**e**) Simulated images of the enhanced fields between Au-NP and Au-film in a sample with an SLG spacer. (**c**,**f**) Electric field profiles near the proximity region along the surface of a sample with an SLG spacer.

To further demonstrate the presence of plasmon tunneling, Fig. 4a compares the QCM and CEM simulations. Shown in the upper and lower rows of the panels in Fig. 4 are the simulation results for 633 nm and 785 nm excitations, respectively. Both QCM and CEM simulations yield almost the same enhancement for samples with a multi-layer graphene spacer (i.e., 2–4 layers). For a sample with an SLG spacer, however, the field enhancement simulated by the QCM is significantly smaller than that obtained by the CEM because the QCM simulation allows a channel for quantum tunneling through the spacer whereas the CEM simulation does not. Figure 4b shows electric field profiles in the region near the SLG spacer when illuminated by a 633 nm laser. The image obtained by the CEM shows highly enhanced near-fields, while that obtained by the QCM shows suppressed field enhancement when compared to the CEM. This can also be clearly seen in Fig. 4c where the magnitude of the electric field is plotted as a function of position along the air-spacer interface. Such a reduction in electric field magnitude is again induced by quantum tunneling. In the case of excitation with a 785 nm laser (Fig. 4d), the difference in the field enhancement between the QCM and CEM remains because of the quantum tunneling in the QCM. However, the amount of difference is significantly less compared to the 633 nm case, mainly because of a relatively weak resonance of plasmonic coupling with this wavelength of light. As discussed above, the difference in the field enhancement between the QCM and CEM disappears as the excitation wavelength increases. The electric field profiles in the region near the SLG spacer and the position-dependent electric field along the surface are shown in Fig. 4e,f.

Lastly, we comment on an optimal thickness of the graphene spacer to obtain a high field enhancement by mentioning the concentration split of BCB in the structure studied here. The double-layer spacer was found to yield the highest enhancement both in the experiment and in the simulation as shown in the Fig. 2. In order to achieve the highest possible field enhancement in the region near a graphene spacer associated with plasmonic coupling such as SERS, a double-layer graphene spacer is desirable because it offers the smallest gap distance that can avoid plasmon tunneling. In a sample with a double-layer spacer, we were able to detect BCB concentrations as low as 1 nM by observing a clearly visible main peak at 581 cm^−1^, as shown in Supplementary Fig. 7a. To check the sample uniformity, Raman signals at 15 randomly selected points in the entire sample area were examined as shown in Supplementary Fig. 7b. The consistent signals indicate homogeneity, which can facilitate uniform plasmonic coupling over the entire area of the double-layer spacer.

## Conclusion

We have investigated plasmonic coupling and the quantum tunneling in a vertically stacked structure of Au-NPs/spacer/Au-film where the thickness of the spacer could be adjusted by the number of graphene layers. One noticeable feature among our observations is that a single-layer graphene spacer is so thin that the near-field enhancement associated with the plasmonic coupling is somewhat suppressed by the presence of quantum tunneling through the spacer. For such a thin spacer, as the gap narrowed, both the experiment and the QCM simulation showed a sudden drop in the field enhancement, whereas the CEM simulation simply showed the conventional exponential increase. Such a discrepancy between the QCM and CEM simulations suggested the presence of quantum tunneling in the case of the SLG spacer.

We also found that the sudden drop in the field enhancement did not occur when the wavelength of incident light did not match the wavelength that induces resonance. When the number of spacer layers was more than two, the CEM and QCM simulations yielded essentially the same results. In the SLG, however, the two simulations showed entirely different behavior. The CEM results still showed a resonance peak, whereas the QCM result did not show any peak in the range of 500–800 nm. This resonance breaking was attributed to plasmon tunneling through the SLG spacer. In the QCM simulation, the field enhancements for a single- and a double-layer graphene spacer were reversed at a wavelength of approximately 700 nm. For this reason, the enhancement for the 633 nm illumination showed a sudden drop, whereas that for the 785 nm illumination continued to increase as the wavelength decreased. As the wavelength became larger, the discrepancy between the enhancements determined by the CEM and QCM simulations became smaller, meaning that the plasmon tunneling did not play a role with the longer wavelengths.

The optimal spacer thickness for obtaining the highest possible field enhancement was found to be a double-layer of graphene, at which a homogenous BCB concentration of 1 nM could be detected throughout the entire area of the sample. This highly resolved measurement of the field enhancement elucidated an aspect of the quantum plasmonics associated with ‘hot spots’ in coupled noble-metal NPs. Although a double-layer of graphene was thought to be an outstanding spacer for the plasmonic coupling in our stacked structure, this thickness may not be appropriate in other structures with different stacking configurations. Furthermore, finding an optimal thickness of a spacer made of materials other than graphene is an entirely different problem. One may further pursue to resolve such a fascination feature of plasmonic coupling in a spacer structure by attempting to use other two-dimensional materials such as hexagonal boron nitride (h-BN).

## Experimental Section

### Graphene synthesis

Inductively coupled plasma chemical vapor deposition (ICP-CVD) was used to synthesize SLG films. A copper thin film (Cu) with a thickness of 300 nm was first deposited on a silicon dioxide wafer by an electron beam evaporator. The wafer was then loaded in a chamber and then annealed by raising temperature up to 800 °C under an inert atmosphere of argon (Ar). To prevent oxidation of the Cu surface, hydrogen gas (H_2_) was injected into the chamber. The Cu film was next exposed to C_2_H_2_ gas for two minutes for graphene growth. Finally, the chamber was cooled down to room temperature under the Ar atmosphere.

### Fabrication details

The bottom gold film of thickness 60 nm was first deposited on a silicon dioxide (SiO2) substrate with a chromium adhesion layer (thickness 10 nm) by a thermal evaporator (KVE-T2010, KVT) under a high vacuum condition (~5 × 10^−6^ torr). The SLG was transferred by the dry transfer method, which had been previously reported by our group^31^. To obtain multi-stacked graphene layer, such a transfer process was repeated. Gold nanoparticles (top Au-NPs) were then deposited on top of the graphene layer by using the same evaporator. Especially in fabricating Au-NPs, the deposition rate was maintained at 0.1 Å s^−1^ and its final thickness was just 3 nm. In order to obtain a nearly spherical shape of NPs, the substrate was annealed at about 350 °C for 1 hour under the Ar atmosphere^32^. Finally, BCB droplets of a concentration of 10^−4^ M were dropped on top of the sample surface, and then the sample was dried in air.

### Simulation details

Both the QCM and CEM simulations were performed by using a commercial simulation package (COMSOL Multiphysics). A graphene spacer layer was sandwiched between a bottom gold film and top gold nanoparticles. Due to required huge calculation resources and slow convergence of a three-dimensional simulation, we perform two-dimensional simulation which assume Au-NP as infinitely long cylindrical structure. For simplicity, a chromium adhesion layer below the gold film and a silicon oxide substrate used in the experiment were ignored. Input parameters used were as follows: the diameter of Au-NPs is 24.1 nm which was the average value obtained from SEM images. Both Au-NPs and Au film were assumed to be described by the Drude model with ε_m_ = 1, ω_p_ = 9.065 eV, γ_p_ = 0.0708 eV^33^. The spacer thickness can be determined by the number of layers multiplied by the SLG thickness (0.34 nm). The work function of graphene was assumed to be 4.6 eV. To directly apply conventional quantum corrected model, the graphene spacer is modeled as 0.34 nm thickness anisotropic lossy dielectric layer. The in-plane permittivity is derived from the optical sheet conductivity of graphene^34^, and the out-of-plane permittivity is set to 9^35^. A normally incident plane wave was assumed to be linearly polarized in the direction parallel to the sample surface. For a detailed map of field intensities near the spacer gap, the structure near the gap was arranged to operate with fine meshes, each with a spacing of 0.1 nm. The field enhancement, which is considered as the magnitude of SERS, can be given by the maximum value of |E/E_0_|^4^ above the graphene region. The reason for restricting such a region of fields in the enhancement calculation lies in the fact that BCB molecules are finally placed on the graphene in the experiment. Especially in QCM-based simulations, the QCM channel was introduced between Au-NP and Au film. Such a channel allows the calculation to include the quantum tunneling and the tunneling resistance through the gap^26^ (See Supplementary Fig. 1). The width of the QCM channel was 20 nm which is enough to cover the tunneling region. The effective plasma frequency used was the same as that used for the classical simulation. However, the permittivity was modified as follows:

$${{\epsilon }}_{g}(\omega ,l)={{\epsilon }}_{0}(\omega )+({{\epsilon }}_{m}(\omega )-{{\epsilon }}_{0}(\omega )){e}^{-l/{l}_{d}}-\frac{{\omega }_{p}^{2}}{\omega (\omega +i{\gamma }_{p}{e}^{l/{l}_{c}})}$$

where *l* is the gap distance between Au-NP and gold film. The plasma frequency *ω*_*p*_ and scattering rate ${\gamma }_{p}$ of gold were used as parameters in effective medium. The *∈*_*o*_(ω) is the permittivity of dielectric layers. The tunneling strength of QCM channel is described by the parameter *l*_*c*_ = 0.0589 nm which is derived from the tunneling of the conduction electron for the graphene potential wall. The tunneling current is calculated for a parabolic shape potential which includes reducing of potential barrier by image charge. Based on Drude model and Ohm’s law, the permittivity of QCM channel is obtained from the conductivity of tunneling current. The contribution of the localized *d*-electrons which decays exponentially with the increasing gap distance, is introduced by ${l}_{d}=0.079\,{\rm{nm}}$^33^.

### Structural characterizations and Raman spectroscopy

The images of Au-NPs were obtained by using a scanning electron microscope (SEM) (Magellan 400, FEI). The average diameter of NPs and the interparticle distances were characterized by using an image processing software, ImageJ^36^. The cross-section TEM images were obtained by using a Cs-corrected Titan TEM instrument (FEI). To prevent possible damages during the process of focused ion beam (FIB), an aluminum oxide (Al_2_O_3_, thickness of 40 nm) protecting layer was formed on the top of Au-NPs by using atomic layer deposition (ALD). The SERS measurement was carried with a high-resolution dispersive Raman spectrometer (ARAMIS, Horiba Jobin Yvon) with the excitation wavelength of 633 nm and 785 nm. A 50× objective (NA 0.75) was used to focus the laser beam and to collect the Raman signal. The size of laser spot is approximately 1 µm. The Raman intensities were averaged over 5–15 points randomly selected in the entire area of the sample. Data were taken with integration time of 30 seconds between 300 and 1250 cm^−1^. Brilliant cresyl blue (BCB, Sigma-Aldrich) was used as SERS test molecules.

## Supplementary information

Supplementary information
