# Supplementary material for: Observation of Wavelength-Dependent Quantum Plasmon Tunneling with Varying the Thickness of Graphene Spacer
Source: Sci Rep. 2019 Feb 4;9:1199. doi: 10.1038/s41598-018-37882-z (PMC6362230; doi:10.1038/s41598-018-37882-z)
Supplement: Supplementary file 1 — Supplementary information [file 41598_2018_37882_MOESM1_ESM.docx]

Observation of Wavelength-Dependent Quantum Plasmon Tunneling with Varying the Thickness of Graphene Spacer

*Khang June Lee,^†^ Shinho Kim,^†^ Woonggi Hong, Hamin Park, Min Seok Jang,*

*Kyoungsik Yu,* and Sung-Yool Choi**

School of Electrical Engineering, Center for Advanced Materials Discovery towards 3D Displays, Korea Advanced Institute of Science and Technology (KAIST), 291 Daehak-ro, Yuseong-gu, Daejeon, 34141, Republic of Korea

†K. J. Lee ([plamingo07@kaist.ac.kr](mailto:plamingo07@kaist.ac.kr)) and †S. Kim (kimsinho6890@kaist.ac.kr) contributed equally to this work.

*Address correspondence to S. Y. Choi ([sungyool.choi@kaist.ac.kr](mailto:sungyool.choi@kaist.ac.kr)) and K. Yu (ksyu@kaist.ac.kr)


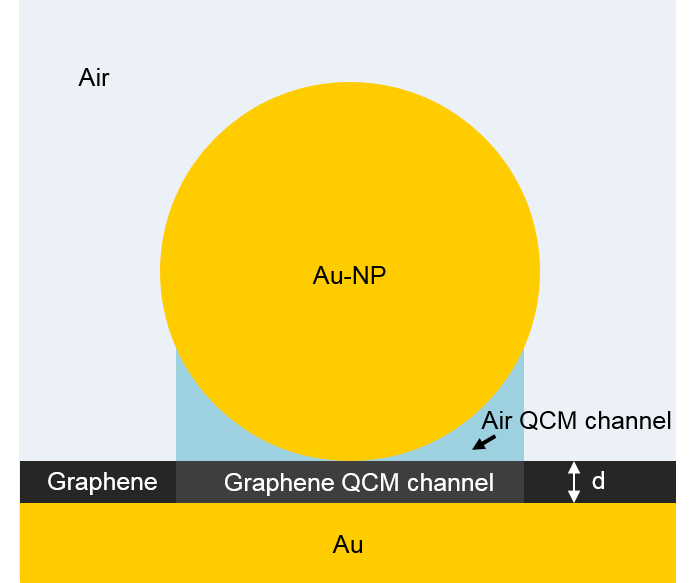


Figure S1. Schematic illustration of the FEM-based electromagnetic simulation model considering the QCM effects. A spherical Au-NP with a diameter of 24.1 nm is placed on the surface of a graphene, which is in contact with a flat Au film. A QCM channel of horizontal width 20 nm is introduced between Au-NP and Au-film. As in the experiments studied here, a plane wave is incident normally on the sample and excites the plasmonic coupling. See the Method section for details.


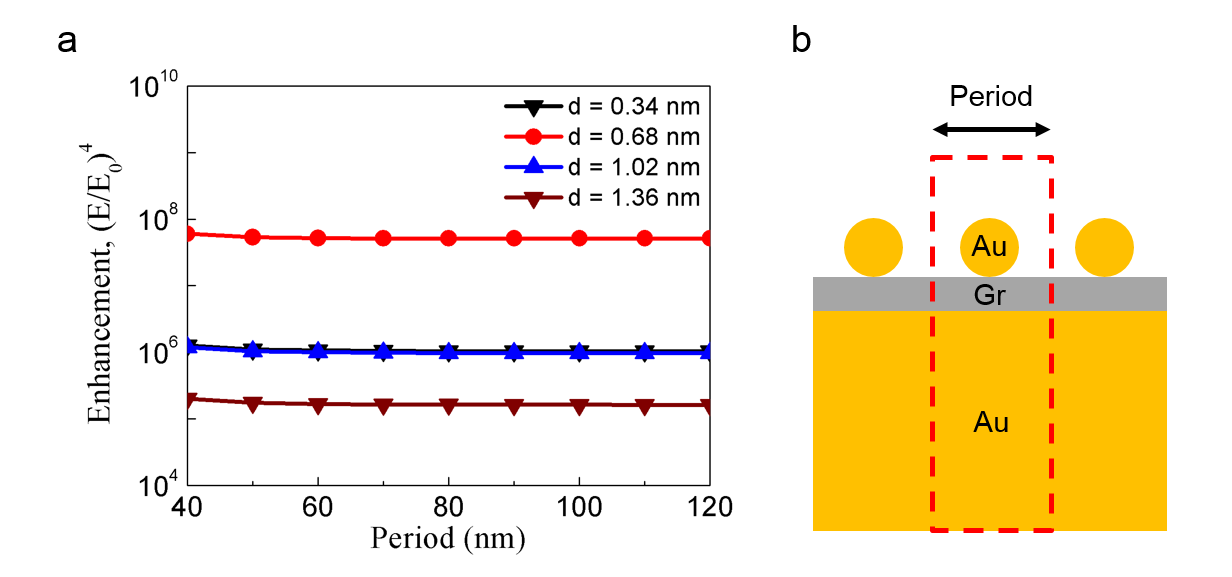


Figure S2. Simulated enhancement as a function of period (i.e., the inter-particle distance between NPs). The simulation results were obtained by setting the periodic boundary conditions for approximating a large system of the NPs/graphene/film structure. Note that two curves with different thicknesses of 0.34 nm and 1.02 nm are almost overlapped over the whole range of periods. The enhancement do not show any meaningful change as the spatial period decreases down to about 40 nm. This implies that the lateral plasmonic coupling may be ignored because of the relatively large separation between adjacent Au-NPs, compared with the corresponding vertical coupling.


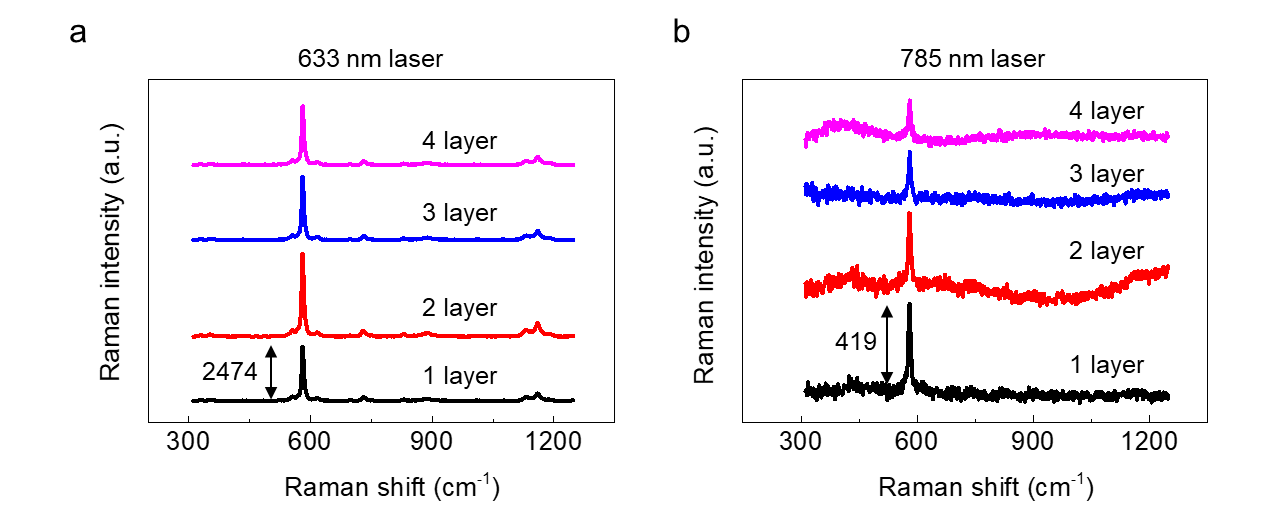


Figure S3. Dependence of the Raman spectra of BCB molecules on the number of graphene layers: (a) with 633 nm laser excitation and (b) with 785 nm laser excitation. In the case of an SLG spacer (black line), the reason that the Raman intensity obtained with 633 nm excitation in much larger than that obtained with 785 nm excitation lies in the fact that the plasmonic resonance occurs near the wavelength of 633 nm. The highest Raman intensity of samples excited by 633 nm laser occurs at double-layer spacer, whereas that of samples excited by 785 nm laser occurs at single-layer spacer.


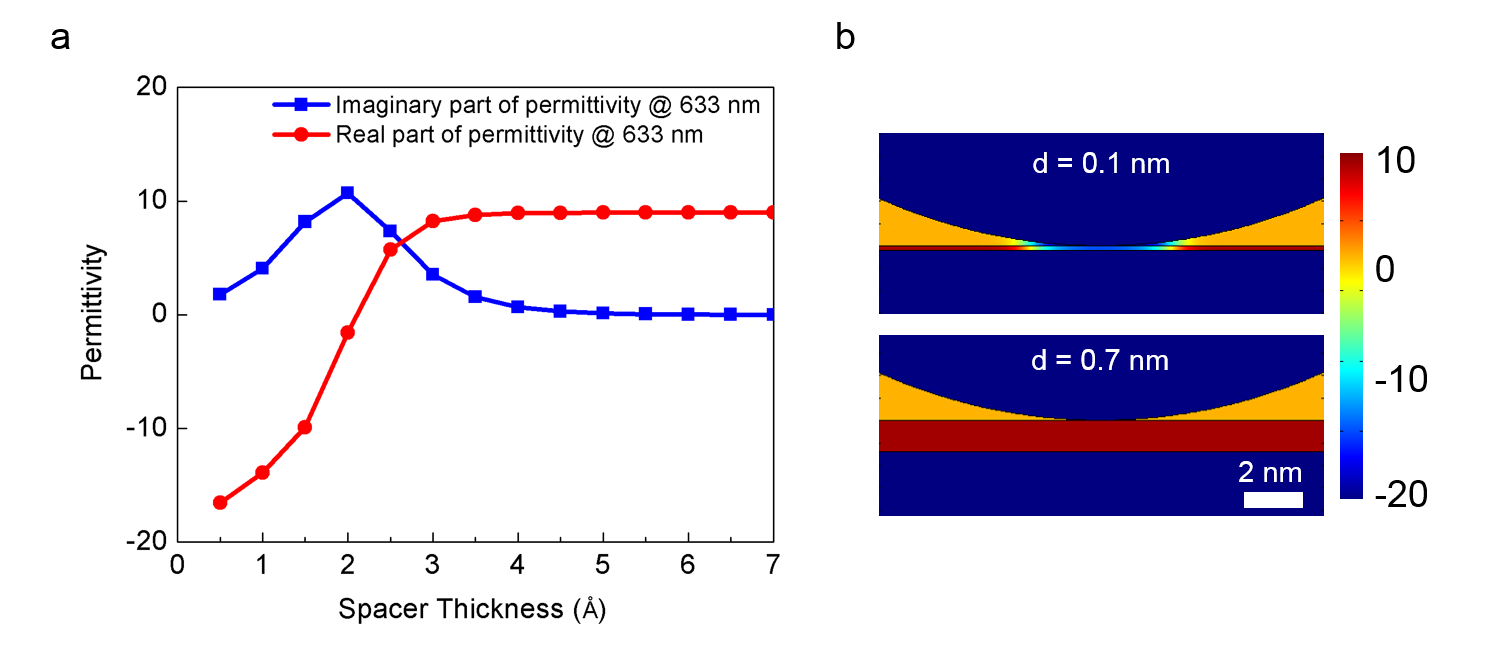


Figure S4. (a) Relative permittivity of the graphene QCM channel sandwiched between NPs and a film as a function of thickness *d*. As the spacer thickness decreases, the real part of the permittivity begins to decrease at about *d*~4 Å and then becomes negative at very small thickness. (b) Simulated images of the relative permittivity for two gap thicknesses: 1 Å (upper panel) and 7 Å (lower panel). The relative permittivity of the 7 Å gap remains constant along the direction of the channels, while that of the 1 Å gap shows negative values near the touching region.


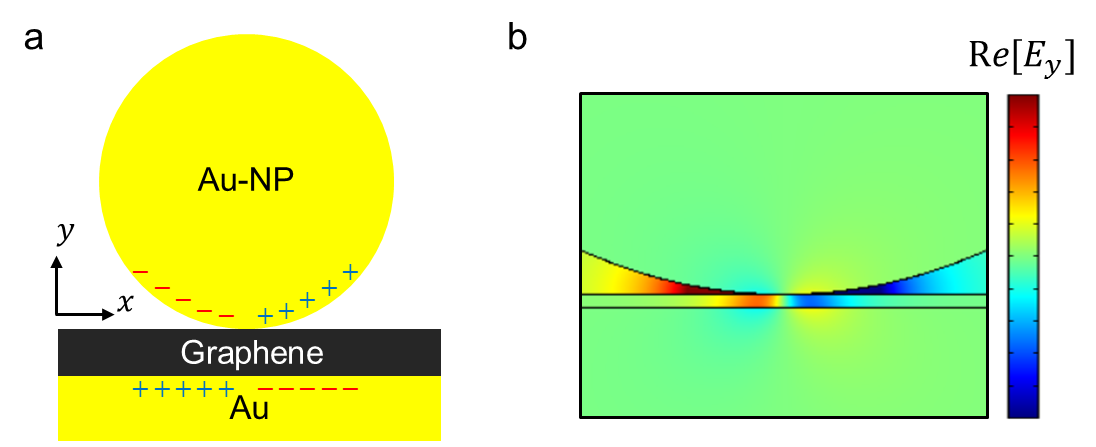


**Figure S5.** (a) The schematics of charge distribution in resonant mode. The red straight bar and blue cross bar indicate negative and positive charges, respectively. (b) The y component of electric field distribution at resonance wavelength (~685 nm).


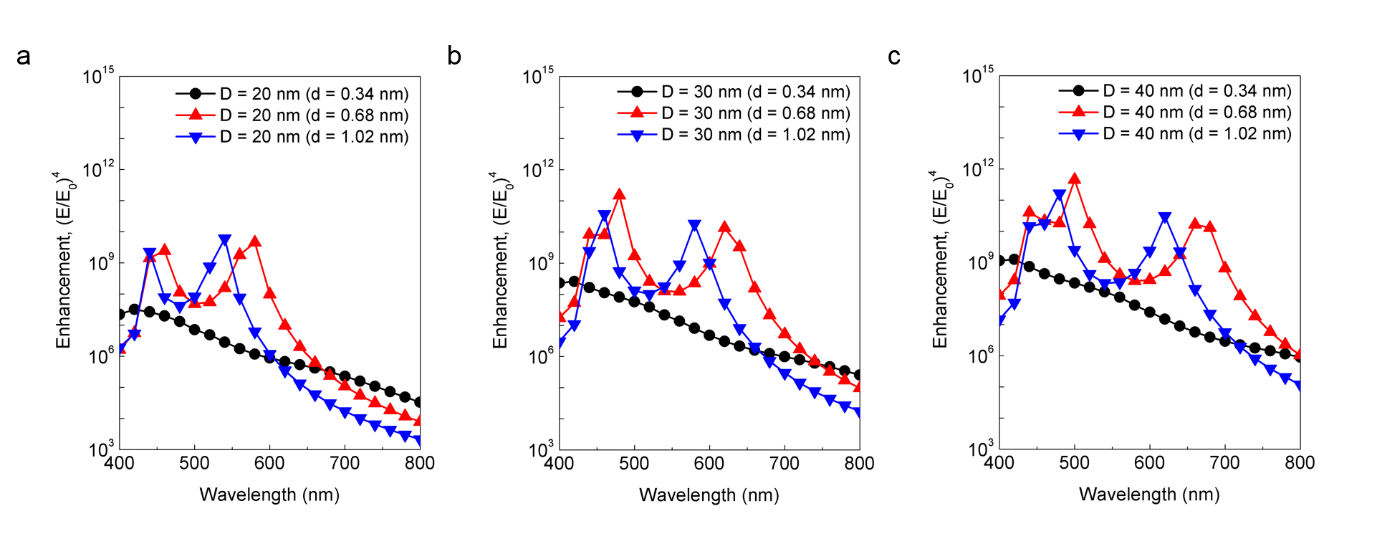


Figure S6. Simulation results of the enhancement as a function of input wavelength. The diameter of nanoparticles used in the simulation was (a) D = 20 nm, (b) 30 nm, and (c) 40 nm. Each panel shows three curves, each corresponding to a different spacer thickness. As the diameter increases, the enhancement increases and the peaks associated with the plasmonic resonance undergo a redshift. The samples with an SLG spacer do not show the resonance peak because of the presence of the plasmon tunneling.


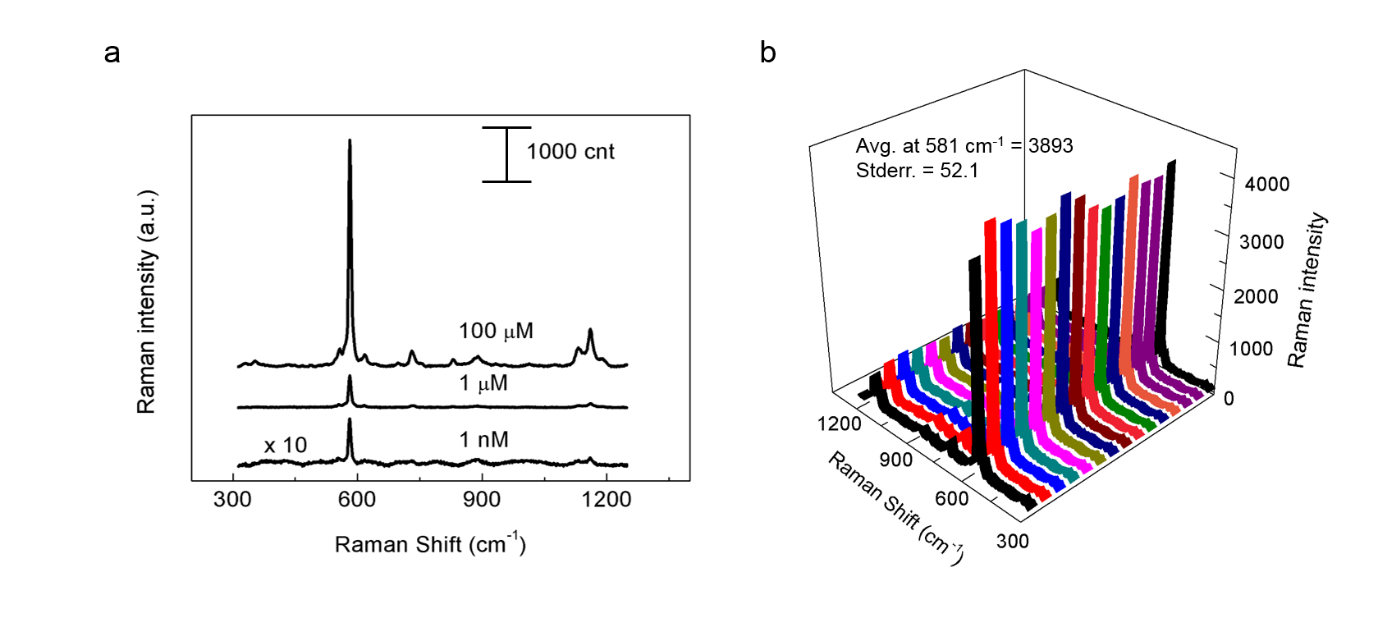


Figure S7. (a) Raman spectra of BCB molecules in the double-layer graphene spacer case. According to our studies, the highest field enhancement was observed in a sample with a double-layer spacer at a resonance wavelength of ~633 nm. The main Raman peak at 581 cm^-1^ is clearly visible even for diluted BCB concentrations as low as 1 nM. (b) Raman spectra measured at 15 different locations randomly selected within the sample area (about 2 cm^2^) show a good homogeneity in the Raman signals. This implies that the double-layer graphene spacer thickness and NP dimensions are uniform over the entire area of the sample.
